# Les neuropathies liées au VIH/SIDA: une étude clinique chez les patients infectés par le VIH au Centre d'Excellence VIH/SIDA de l'Université de Lubumbashi

**DOI:** 10.11604/pamj.2015.20.392.5799

**Published:** 2015-04-21

**Authors:** Joe Katabwa Kabongo, Célestin Kaputu-Kalala-Malu, Oscar Luboya, Valerien Mutombo, Abel Ntambwe, Mala Ali Mapatano, Kavulu Mayamba Mukendi

**Affiliations:** 1 Département de Médecine Interne, Service de Neurologie, Université de Lubumbashi (UNILU), République Démocratique du Congo; 2 Centre Neuropsychopatholgique (CNPP), Département de Neurologie, Service de Neurologie Pédiatrique, Université de Kinshasa (UNIKIN), République Démocratique du Congo; 3 Département de Pédiatrie, Université de Lubumbashi (UNILU), République Démocratique du Congo; 4 Département de Neurologie, Université Officielle de Mbuji-Mayi, République Démocratique du Congo; 5 Département de Santé Publique, Université de Lubumbashi (UNILU), République Démocratique du Congo; 6 Département de Santé Publique Université de Kinshasa (UKIKIN), République Démocratique du Congo; 7 Centre Hospitalier Régional (CHR) Mons-Hainaut (Mons) et cliniques Universitaires Saint-Luc, Université Catholique de Louvain (UCL), Département de Pédiatrie, Cliniques Universitaires de Lubumbashi (UNILU), République Démocratique du Congo

**Keywords:** Neuropathie, HIV, TARV, CD4, Lubumbashi, République Démocratique du Congo, Neuropathy, HIV, TARV, CD4, Lubumbashi, DRC

## Abstract

**Introduction:**

En vue d'améliorer la prise en charge des patients souffrant de neuropathie (NP) associées à l'infection HIV, nous avons essayé de déterminer le profil clinique des personnes souffrant de NP au cours du suivi thérapeutique de leur infection HIV.

**Méthodes:**

Il s'agit d'une étude transversale (n= 101) menée au centre d'excellence depuis 1 an. Notre analyse est essentiellement clinique. Par un examen clinique minutieux, nous avons recherché tous les symptômes et signes cliniques des NP. Subjectivement, les douleurs dominent le tableau. Pour affiner leur diagnostic, nous avons utilisé l’échelle DN4 (Diagnostic des douleurs neuropathiques) et l’échelle EVA (Evaluation de la gravité des douleurs). Nous avons ensuite analysé nos données en fonction de certains autres facteurs épidémiologiques tels que le taux des CD4, le traitement anti-HIV etc.

**Résultats:**

Les 101 patients représentent 3,12% de la cohorte générale; 53,3% des patients présentent une abolition des réflexes ostéotendineux des membres inférieurs; 77,89% présentent une hypoesthésie thermo algique en chaussette et en gants; 25% ont présenté une amyotrophie des membres inférieurs; 76,5% ont été soumis à un traitement antirétroviral contenant la stavudine; 11,7% ont pris la didanosine (DDI) et Abacavir (ABC). 84% ont une moyenne de CD4 de 292 cel/mm^3^.

**Conclusion:**

La NP altère la qualité de vie de nos patients et diminue l'adhérence au traitement antirétroviral. Plusieurs facteurs sont incriminés dans la survenue de la NP, l'effet direct des antirétroviraux, l'effet inflammatoire dysimmunitaire, l'effet infectieux lié aux infections opportunistes. D'autres facteurs seront recherchés et analysés ultérieurement.

## Introduction

Le virus de l´immunodéficience humaine (VIH), qui entre dans sa quatrième décennie de l’épidémie, affecte environ 33 millions de personnes vivant dans les pays développés et dans les pays à ressources limitées. Les complications neurologiques du système nerveux périphérique sont fréquentes chez les patients infectés par le VIH et la pathologie neuromusculaire est associée à une morbidité importante [1]. En pratique, les situations cliniques les plus fréquemment rencontrées sont les suivantes: Les polyneuropathies, caractérisées par une atteinte distale et symétrique, à prédominance sensitive, touchant essentiellement les membres inférieurs. C´est la situation clinique la plus fréquente. Dans ce cas, le diagnostic étiologique se fait entre les causes liées aux médicaments anti HIV et l'action directe du virus [2]; Une sensation de pieds brûlants (burning feet). De nombreuses études soulignent le caractère très prononcé des dysesthésies de contact, de l'allodynie et de la marche antalgique, évitant l'appui trop marqué sur les plantes des pieds hyperesthésiques. Les crampes sont également fréquentes. Des douleurs marquées sont surtout constatées aux stades tardifs, de même que l´association à des signes centraux et à des troubles cognitifs; Les réflexes ostéotendineux sont diminués ou abolis aux membres inférieurs. La coexistence de réflexes achilléens moyens à vifs, d´une hyperréfléxie rotulienne et d´un signe de Babinski doivent faire évoquer une myélopathie associée [2, 3]. Les Polyneuropathies distales sont soit d'origine iatrogène, soit liées directement au VIH. Les premières sont devenues très fréquentes depuis l´utilisation des trithérapies [2, 3] Les trois molécules mises en cause dans l’étiologie des polyneuropathies iatrogènes sont la zalcitabine (ddC ou Hivid), la didanosine (ddl ou Videx) et la stavudine (d4T ou zérit). Il a été décrit des cas de paresthésies péribuccales et des extrémités sous ritonavir (Norvir) et amprénavir (Agénérase), sans neuropathie objective. De rares cas d´atteinte nerveuse périphérique ont été attribués au saquinavir (lnvirase), ainsi qu´un risque d´aggravation d'une neuropathie préexistante sous nelfinavir (Viracept) [2, 4]. L'objectif de notre étude est de déterminer le profil clinique des personnes souffrant de la NP au cours d'une infection HIV en vue d'améliorer leur prise en charge.

## Méthodes

Il s'agit d'une étude transversale de 101 patients sélectionnés au préalable pour des problèmes de neuropathies. Notre étude a été faite au Centre d'Excellence VIH/SIDA de l'Université de Lubumbashi à l'hôpital Sendwe. Le type d’échantillonnage est non probabiliste. Nous avons procédé à un recrutement en boule de neige de toutes les personnes de la cohorte générale qui présentaient la NP. Le diagnostic est essentiellement clinique, les plaintes du patient en faveur de la NP, un examen neurologique minutieux pour rechercher les troubles de la sensibilité superficielle et profonde, les réflexes et la force musculaire des membres affectés. La douleur sera ensuite évaluée par 2 échelles. L’échelle analogique EVA pour l’évaluation de l'intensité de la douleur et L’échelle DN4 pour l'orientation diagnostique des douleurs neuropathiques. Dans l’échelle EVA, on demande au patient de définir le degré de sa douleur sur une réglette graduée de 1 à 10. Le score inférieur à 5 équivaut à une douleur supportable; un score supérieur à 5 équivaut à une douleur importante à insupportable. Dans l’échelle DN4, un questionnaire est soumis aux patients. Un score supérieur ou égal à 4 est en faveur d'une douleur neuropathique [4, 5]. Les patients qui fréquentent le Centre d'Excellence, en ambulatoire, reçoivent un traitement antirétroviral hautement actif (HAART). Nous connaissons leur évolution biologique, immunologique et clinique. Ils ont tous un diagnostic confirmé de VIH/sida. Ils ont plus de 18 ans et capable de fournir un consentement éclairé. Ils parlent couramment le swahili, langue parlée au Katanga et le français, au moment de la récolte des données. Le questionnaire a été traduit dans les deux langues. L´autorisation de procéder à l´étude a été soumise au comité d’éthique de l'Université de Lubumbashi. Un consentement écrit a été demandé à tous les participants. La saisie et l'analyse des données ont été faites à l'aide du logiciel SPSS 11.0 et Epi Info version 3.5.1 et Excel 2010.

## Résultats

**Figure 1:**
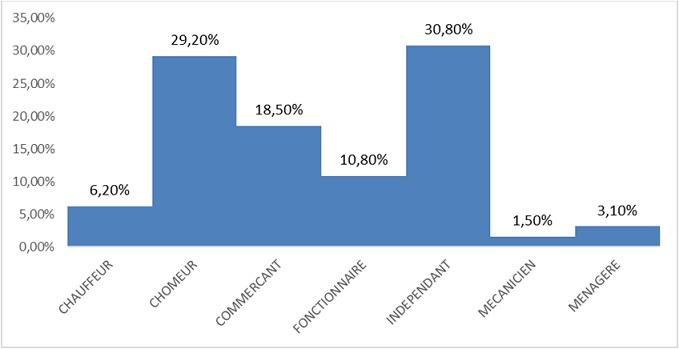
Répartition de l’échantillon selon la Profession

**Figure 2:**
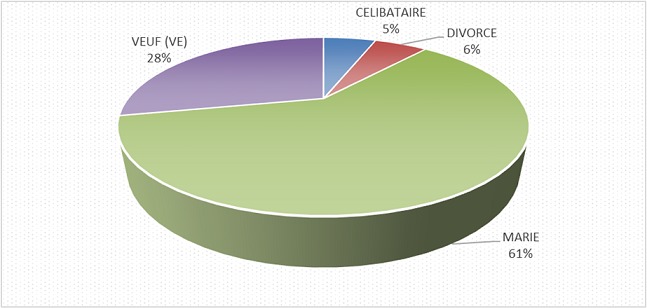
Répartition de l’échantillon selon l'Etat civil

**Figure 3:**
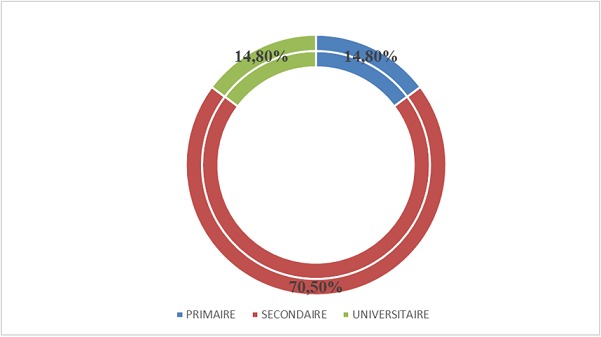
Répartition de l’échantillon selon le niveau d’étude

**Figure 4:**
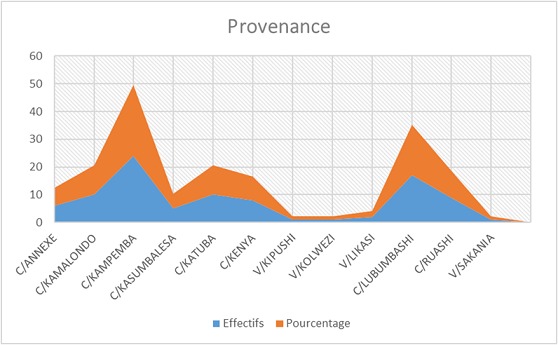
Répartition de l’échantillon selon la Provenance

**Figure 5:**
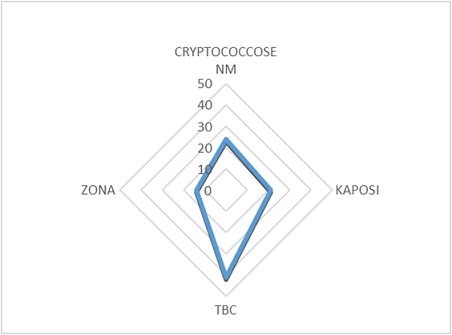
Répartition de l’échantillon selon la survenue des infections opportunistes

Notre étude a porté sur 101 patients soit 3,12% de la cohorte générale. 53,19% sont des femmes, dont 90% ont un âge compris entre 25 et 55 ans; 91% des hommes ont un âge compris entre 46 et 70 ans (Tableau 1), 30% d'entre eux exerce une profession libérale (Figure 1). 61% de nos patients sont mariés (Figure 2). Selon le niveau d'instruction, 70,5% de nos patients ont un niveau d’études secondaires (Figure 3). Nous avons reparti nos patients selon leur provenance et avons constaté que 48% proviennent de la commune de Kampemba, l'une des importantes communes de Lubumbashi et même la plus grande (Figure 4). Nous avons essayé de connaître la part des infections opportunistes dans notre série et nous constatons que 48% de nos patients ont souffert de la tuberculose et ont été traité pour cette affection (Figure 5). Les manifestations cliniques des NP ont été analysées et 53,3% des patients ont les réflexes ostéotendineux abolis, 77% ont la sensibilité vibratoire diminuée et 76,6% ont la sensibilité superficielle diminuée ou absente (Tableau 2). 80% des patients ont consulté pour douleurs des membres inférieurs, 25% ont présenté une amyotrophie des membres inférieurs et environ 3,6% présentent une réelle difficulté à la marche. L'intensité de la douleur a été évaluée à l'aide de l’échelle EVA et 100% de nos patients souffrant de NP ont un score supérieur ou égal à 5 sur 10, ce qui exprime une intensité douloureuse subjective importante à insupportable (Tableau 3). L’évaluation de la douleur neuropathique a été testée à l'aide de l’échelle DN4 et tout score supérieur à 4 est en faveur d'une douleur neuropathique. 94,5% des patients souffrant de la NP ont un score supérieur ou égal à 4 (Tableau 4). Les affections opportunistes les plus fréquentes au cours de l'infection HIV sont retrouvées avec des fréquences variables dans notre étude (Figure 5). Selon le schéma thérapeutique appliqué 70,2% de nos patients ont reçu une triple association thérapeutique faite d'azydothymine, de lamivudine et de nevirapine (Tableau 5).

**Tableau 1:** Répartition de l’échantillon selon l'Age et le Sexe

SEXE				
	N	%	N	%
	FEMININ		MASCULIN	
	N	%	N	%
25-30	2	1,98	0	0
31-35	5	4,95	0	0
36-40	13	12,87	5	4,9
41-45	14	13,86	0	0
46-50	6	5,94	6	5,9
51-55	8	7,92	8	7,9
56-60	4	3,96	12	11,8
61-65	3	2,97	6	5,9
66-70	0	0	9	8,9
	**55**	**54,46**	**46**	**45,54**

Les femmes représentent 54,46% de notre échantillon dont la tranche d’âge la plus touchée est comprise entre 36 et 45 ans

**Tableau 2:** Répartition de l’échantillon selon l’évaluation à l'examen neurologique

	ROT		SPV		SS		FMS		FMG	
	N	%	N	%	N	%	N	%	N	%
ABOLI	54	53,5	0	0	0	0	0	0	0	0
CONSERVEE	20	19,8	22	22	25	24,8	97	96	94	93
DIMINUEE	27	26,7	79	78	76	75,2	4	4	7	7
Total	**101**	**100**	**101**	**100**	**101**	**100**	**101**	**100**	**101**	**100**

ROT: reflexes ostéotendineux; FMS: force musculaire segmentaire; SPV: sensibilité profonde vibratoire; FMG: force musculaire globale; SS: sensibilité superficielle

53,5% des patients ont leurs ROT abolis, 77% ont leur SPV diminuée, 75,2% ont leur SS diminuée

**Tableau 3:** Répartition de l’échantillon selon l’échelle EVA

EVA Cotation	
SCORE	%
**5**	13
**6**	26,1
**7**	30,4
**8**	28,3
**9**	2,8
**Total**	**100**

100% des patients ont supérieur ou égal 5/10

**Tableau 4:** Répartition de l’échantillon selon l’échelle DN4 (Diagnostic des douleurs neuropathiques)

ND4 Cotation	
SCORE	%
**2**	6,5
**4**	28,3
**5**	17,4
**6**	21,7
**7**	23,9
**8**	2,2
**Total**	**100**

93,5% de patients ont un score supérieur ou égal à 4/10 ce qui place leurs douleurs dans la catégorie des douleurs neuropathiques

**Tableau 5:** Répartition de l’échantillon selon le schéma thérapeutique

Schémas	Effectifs	Pourcentage
ABC + DDI + LPV	11	10,89
AZT + 3TC + NVP	73	72,28
DUOVIR + EFZ (AZT + 3TC + EFZ)	6	5,94
TDF + 3TC + EFZ	9	8,91
TDF + 3TC + LPV	2	1,98
Total	101	100

72,28% de nos patients sont sous azydothymine (AZT); lamivudine (3TC); nevirapine (NVP)

## Discussion

Dans une population de personnes souffrant d'HIV et bénéficiant de médicaments antiviraux hautement actifs, la survenue de neuropathies est un fait courant. 3,12% de patients suivis dans notre centre pour HIV souffrent de neuropathies. Cette affection est la complication de plusieurs facteurs retrouvés dans les infections HIV. Comme on l'a signalé dans l'introduction, les neuropathies remarquées dans notre série peuvent être liées à l'action directe du virus HIV, à la complication neuropathique des médicaments antirétroviraux ou aux effets dysimmunitaires concomitants à l'infection HIV. Il est difficile de confirmer avec exactitude l'une de trois hypothèses évoquées ci-dessus, surtout dans notre milieu où les moyens techniques complémentaires font défaut. Des facteurs individuels constitutionnels peuvent être évoqués d'autant plus qu'il n'y a que 3,12% de patients suivis en traitement anti HIV qui souffrent de NP. Nous n'avons pas non plus analysé les facteurs liés à d'autres types de toxiques susceptibles de provoquer des NP chez des patients souffrant d'HIV tels que l'abus de l'alcool, les facteurs vasculaires, le diabète, le tabagisme et l'abus des drogues. L'association de ces facteurs à l'HIV augmente sensiblement la survenue de neuropathies. Voir entre autres Robinson et al. [6]. Il est possible que la dose élevée des médicaments antirétroviraux soit responsable de la survenue de neuropathies chez des patients souffrant de l'HIV comme le signale Simpson D.M. et al [7], mais aucun de nos patients n'a reçu une dose supérieure à celle mentionnée dans cette étude. Cet aspect mérite d’être exploré dans une autre étude. La douleur neuropathique constitue le symptôme le plus fréquent au cours des neuropathies quelque soient leurs étiologies. Elle altère la qualité de vie des patients qui en sont atteints. Dans notre étude, 100% de nos patients présentent une intensité de la douleur supérieure à 5 sur l’échelle EVA; ce qui correspond à une douleur insupportable à intolérable. Ceci a été rapporté par certains auteurs [8, 9]. Parmi les affections opportunistes qui surviennent au cours de l'infection HIV, la tuberculose est l'affection la plus rencontrée en Afrique subsaharienne. Parmi nos patients souffrant de la NP, 40% ont souffert de la tuberculose. L'effet du traitement antituberculeux est incriminé dans la survenue de la NP [8, 9]. Une importante population (40%) des patients souffrant de NP s'est présentée à la consultation pour la toute première fois avec un taux de CD4 inférieur à 500 cel/mm; Certains auteurs ont établi une corrélation entre ce taux bas des CD4 et la survenue de la NP [8]. Ceci mérite d’être vérifié dans une étude ultérieure. Nous constatons que 70,2% de patients souffrant de la NP prennent une association thérapeutique comprenant l'azydothymine (AZT), la lamivudine (3TC) et la nevirapine (NVP). Les antirétroviraux sont incriminés dans la survenue de la NP chez des patients HIV. Ceci a été également constaté par certains auteurs dans plusieurs autres associations des médicaments antirétroviraux et ce fait attire notre attention [9–15].

## Conclusion

La morbidité croissante des NP chez les patients souffrant d'HIV altère leur qualité de vie et diminue la compliance vis-à-vis de leur traitement antirétroviral. 3,12% de notre cohorte présente des neuropathies dont la principale plainte est la douleur. Parmi les facteurs incriminés, les ARV viennent en tête, ensuite les affections opportunistes, l'effet direct de l'infection VIH et les probables réactions dysimmunitaires liées à l'infection HIV. Mais beaucoup d'autres facteurs peuvent être recherchés dans la survenue des neuropathies chez des patients HIV sous traitement. Nous pensons que ce phénomène est multifactoriel et il mérite d’être approfondi par d'autres explorations cliniques, éléctrophysiologiques (ENMG) et neuropathologiques (Biopsies neuromusculaires). Tous les facteurs collatéraux doivent également être explorés. La prise en charge adéquate de cette affection doit être l'une des priorités dans les mesures sociales à prendre pour le suivi de ces patients.
